# Hughes’ Questions on Prosthetic Dentistry

**Published:** 1914-12-15

**Authors:** 


HUGHES' QUESTIONS ON PROSTHETIC DENTISTRY. This is a little book by Dr. C. N. Hughes, of Atlanta, stating questions and giving the answers. It is designed for class reviews and as the author says, to prepare for State Board Examinations. There is some question as to the educational value of such books, as too often the student depends upon them for his knowledge, and should his memory be faulty he fares badly when the test comes. This book may be needed, but we could give more satisfactory answers to many of the questions, and the questions in many cases are too elementary and catagorical. For instance: "What is the purpose of an impression tray?" Answer: "To hold impression material while introducing into the mouth and hold in shape while preparing model." Or this one: "Which is preferable, plate or bridge?" Answer: "Bridge." Of course it is not all like this, but there is this tendency to incompleteness and educational development too conspicuous and persistent. It is published by the C. V. Mosby Co., St. Louis.

